# A case report and literature review of a rare lymphoma with repeated fever by pain and numbness in both lower limbs

**DOI:** 10.3389/fmed.2025.1629134

**Published:** 2025-08-06

**Authors:** Xiaoqiong Duan, Wenhua He, Rong Chen, Min Jing, Xin Wang

**Affiliations:** ^1^Department of Hematology, Suining Central Hospital in Sichuan Province, Suining, China; ^2^Medical Imaging Department of Suining Central Hospital in Sichuan Province, Suining, China; ^3^Sichuan Jinyu Medical Inspection Center Chengdu, Chengdu, China

**Keywords:** IVLBCL, IL-10, BTK inhibitor, CNS invasion, CES, Orelabrutinib

## Abstract

**Objective:**

Explore the clinical characteristics, diagnostic and therapeutic strategies, and prognosis of patients with intravascular large B-cell lymphoma (IVLBCL).

**Methods:**

Through retrospective analysis of the clinical data of a patient with IVLBCL successfully treated in our department, and in combination with a review of relevant literature, we explore the pathogenesis, diagnostic methods, treatment strategies, and prognostic features of this disease.

**Conclusion:**

IVLBCL as a rare subtype of extranodal large B-cell lymphoma, is characterized by high malignancy, strong invasiveness, and poor prognosis. Currently, there is no consensus on the understanding of this disease, and diagnostic and treatment standards are not yet standardized. Diagnosis primarily relies on pathological and immunohistochemical examinations. In this study, the R-CHOP regimen combined with high-dose MTX and orelabrutinib was used to treat IVLBCL patients with central nervous system involvement, achieving good therapeutic effects. However, due to the limited number of cases and the lack of large-scale clinical validation, the efficacy of this treatment regimen still requires further evaluation.

## 1 Introduction

Intravascular large B-cell lymphoma (IVLBCL), which was first reported in 1959 by Pfleger and Tappeiner as “systemic angioendotheliomatosis of the skin” (1), has an incidence rate of around 0.5 per million. Based on clinical manifestations, the 2022 World Health Organization’s 5^th^ Edition Classification of Tumors of Hematopoietic and Lymphoid Tissues categorizes IVLBCL into the classic, cutaneous, and hemophagocytic subtypes (2, 3). Flaws in IVLBCL cell transvascular migration and homing mechanisms mainly restrict its growth to the inner lining of blood vessels, causing different clinical manifestations and adverse outcomes compared with other lymphomas (4). It is highly clinically and radiologically insidious. Because IVLBCL does not form solid tumors and it rarely affects lymph nodes, it is extremely challenging to detect lesions, which frequently leads to misdiagnosis and missed diagnosis. This case report and literature review aim to enhance our comprehension of this rare disease. We also explore the potential mechanisms by which the combination of orelabrutinib attains a relatively favorable therapeutic effect in treating central nervous system (CNS)-involved lymphoma, thus offering valuable clinical practice perspectives.

## 2 Case presentation

This case involves a 47-year-old male who, in July 2023, experienced progressively worsening pain in the calves of both lower extremities without an obvious cause. Numbness in both lower extremities was also noted in August 2023, along with a loss of urination control. On September 21^st^, 2023, the patient completed examinations at our hospital’s outpatient clinic in the Department of Neurology. Routine blood tests revealed a hemoglobin level of 87 g/L, a lactate dehydrogenase (LDH) level of 1,032 U/L, and an ALB level of 24 g/L (reference ranges: 130–175 g/L, 110–295 U/L, and 39–54 g/L, respectively). Electromyography showed no obvious abnormalities. Regarding routine cerebrospinal fluid (CSF) tests, Pandy’s test for protein was positive (+). A biochemical CSF test revealed a protein quantity of 2.04 g/L (reference range: 0.15–0.45 g/L). Bone marrow (BM) cell morphology, BM cell flow examination, and BM biopsy revealed no abnormalities. Plain MR scan of the head and cervical and thoracic spine on September 23, 2023th: cerebral infarction in the right pons, splenium of corpus callosum, and left centrum semiovale (subacute phase). The patient was given symptomatic pain relief treatment, but the effect was not good. The patient lost weight progressively, losing about 10 kg within 6 months, and numbness and pain in the lower extremities gradually worsened and were accompanied by difficulty in walking.

On October 10^th^, 2023, the patient went to the Department of Neurology at a large Grade A-level hospital in Chengdu, Sichuan Province. He underwent a lumbar puncture test, and CSF biochemistry revealed trace protein (2.98 g/L). Routine CSF analysis revealed nucleated and red blood cell counts of 10 × 10^6^/L and 270 × 10^6^/L, respectively. A tuberculosis antibody test was negative, and the CSF IgG synthesis rate was 65.164 mg/day. CSF smear examination did not detect fungi or bacteria, and ink staining of the smear did not reveal Cryptococcus. Synchronized blood sugar, sodium, and potassium chlorine analysis in the CSF revealed a blood glucose of 6.60 mmol/L and no sodium and potassium chlorine abnormalities. CSF immune cell phenotype analysis did not detect obvious abnormal cell population phenotypes. Fluid-based pathological CSF diagnosis did not find malignant cells. A CSF tuberculosis antibody test was negative. A blood interferon-γ release test for tuberculosis-infected T cells was positive. The ferritin level was 1405.00 ng/ml (reference range: 27–375 ng/ml). Enhanced magnetic resonance imaging (MRI) scans of the cervical and thoracic spine showed significant spinal meningeal enhancement around the conus medullaris and cauda equina, suggesting the need to exclude spinal meningitis around the conus medullaris and cauda equina. For treatment, methylprednisolone (1,000 mg) was administered as a pulse therapy for 4 days. Meanwhile, an anti-tuberculosis trial treatment with isoniazid + rifampicin + ethambutol + pyrazinamide was given. After the treatment, the patient’s body temperature improved, pain in both lower extremities got better, and limb numbness and weakness did not worsen further. On October 23^rd^, 2023, the patient’s condition improved, and he was discharged from the hospital. Outside the hospital, the patient took medication as prescribed by the doctor for 1 month. Later, because of his improved condition, the patient stopped taking the anti-tuberculosis drugs on his own, without seeking advice.

On February 14^th^, 2024, the patient was hospitalized in the Department of Gastroenterology of Suining Central Hospital because of “shortness of breath and heart fatigue for half a month.” On February 19^th^, 2024, the patient was transferred to the Department of Hematology of Suining Central Hospital because of “anemia and fever.” After admission, the attending physician inquired and determined that the patient had had intermittent fever since disease onset, with his body temperature ranging from 38–40°C. Upon transfer, physical examination revealed a peripheral capillary oxygen saturation of 90–91% (with nasal cannula oxygen inhalation at 2–3 L/min). The patient had an acute-illness appearance and an emaciated build. There are no abnormal signs such as maculopapular rash, purpura, etc. on the skin and mucous membranes of the whole body. There was no superficial palpable lymph node enlargement. Breath sounds were relatively low in both lungs. Muscle strength testing revealed a muscle strength of Grade 5 in both upper extremities and Grade 3 in both lower extremities, and both lower extremities had low muscle tone. Upon transfer, the patient’s general condition was poor, and he had low spirits, poor appetite, and irritability. The Eastern Cooperative Oncology Group performance status score was 4.

**Figure 1:**
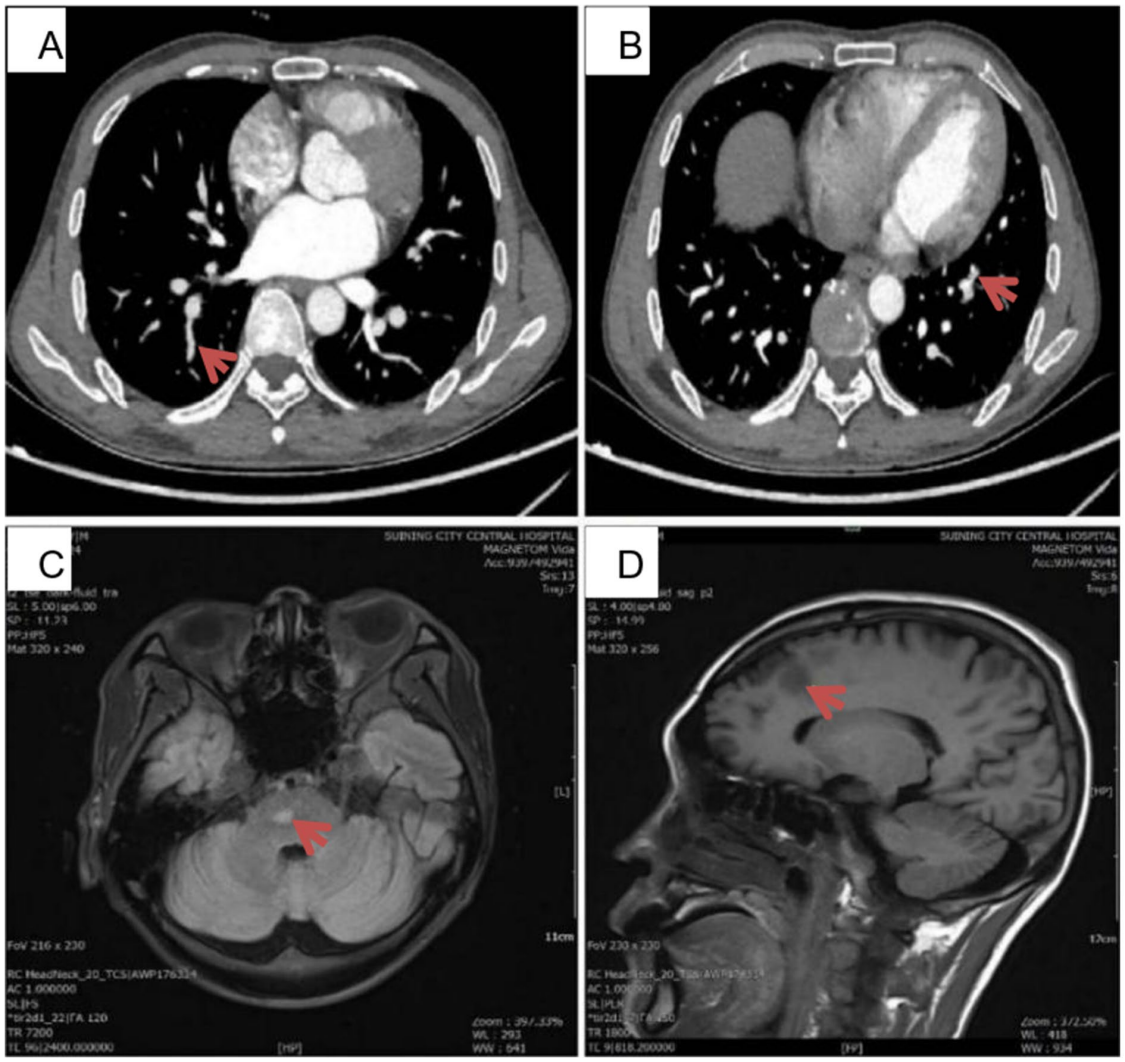
**(A,B)** The patient’s pulmonary artery CTA images on February 20, 2024. In the lower lobes of both lungs, multiple pulmonary arteries are not clearly shown, and filling defect areas can be seen. The posterior basal segment artery of the right lower lobe is shown in **(A)** (red arrow). The anterior medial basal segment artery of the left lower lobe is shown in **(B)** (red arrow), with filling defects. **(C,D)** The plain and enhanced MRI scans of the head and whole spinal cord taken in our hospital on February 29^th^, 2024. There are newly emerged abnormal enhancement foci in the right frontal lobe, the centrum semiovale, and the pons. The red arrow in **(C)** shows the patient’s abnormal lesion at the pons level. The red arrow in **(D)** shows the patient’s abnormal lesion at the right frontoparietal lobe level.

On February 20^th^, 2024, pulmonary artery computed tomography angiography showed: 1. In the lower lobes of both lungs, there seemed to be a few filling-defect shadows in the pulmonary artery branches, with a possibility of pulmonary embolism (Figures 1A,B). On February 22^nd^, another lumbar puncture was carried out at our department. For the CSF routine tests, the Pandy’s test for protein was (+ −). The CSF biochemical test revealed a protein quantity of 1.03 g/L. The Epstein–Barr virus DNA level in peripheral blood mononuclear cells was 1.3E+03 copy/ml (reference range: <5.0E+02 copy/ml). CSF next-generation sequencing revealed an Epstein–Barr virus sequence number of seven and a coverage rate of 0.55% (the reference range is negative). The low coverage and small number of sequences may cause false positive results from background contamination. Plain and enhanced head and whole spinal cord MRI scans were compared with the previous head and thoracic spine films taken on September 22^nd^, 2023. Results suggest that there are new abnormally enhanced foci in the right frontal lobe, central hemiluminal cavity and bear, but it is not clear whether they are caused by infection or cerebral infarction (Figures 1C,D). The patient mainly presented with fever (body temperature: 38–40°C), limb pain and numbness, difficulty in walking, hypoproteinemia, anemia, CNS involvement, hypoxemia, and significantly elevated LDH and ferritin levels. Multidisciplinary team focused on discussion: patients suffer from repeated fever, limb pain and numbness, difficulty walking, accompanied by multi-system damage (nervous system, lungs, urinary system), cerebrospinal cord lesions, hypoxemia, etc. The patient has been healthy in the past, and has no family genetic history. The examination results indicated anemia, thrombocytopenia, albumin reduction, and significantly increased LDH and ferritin. The discussion results and diagnostic considerations were intravascular large B-cell lymphoma or Vascular inflammation.

**Figure 2:**
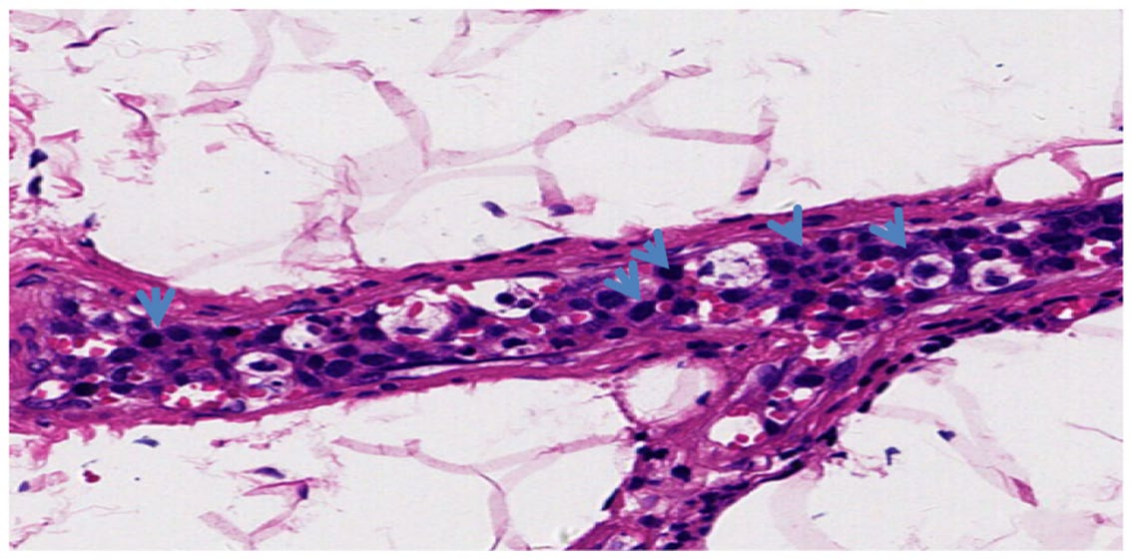
Blood vessel hematoxylin and eosin staining (20X) revealed a large number of tumor cells (blue arrows) inside the blood vessels. The tumor cells were large and loosely arranged, with vesicular nuclei, clear nucleoli, and numerous mitotic figures.

**Figure 3:**
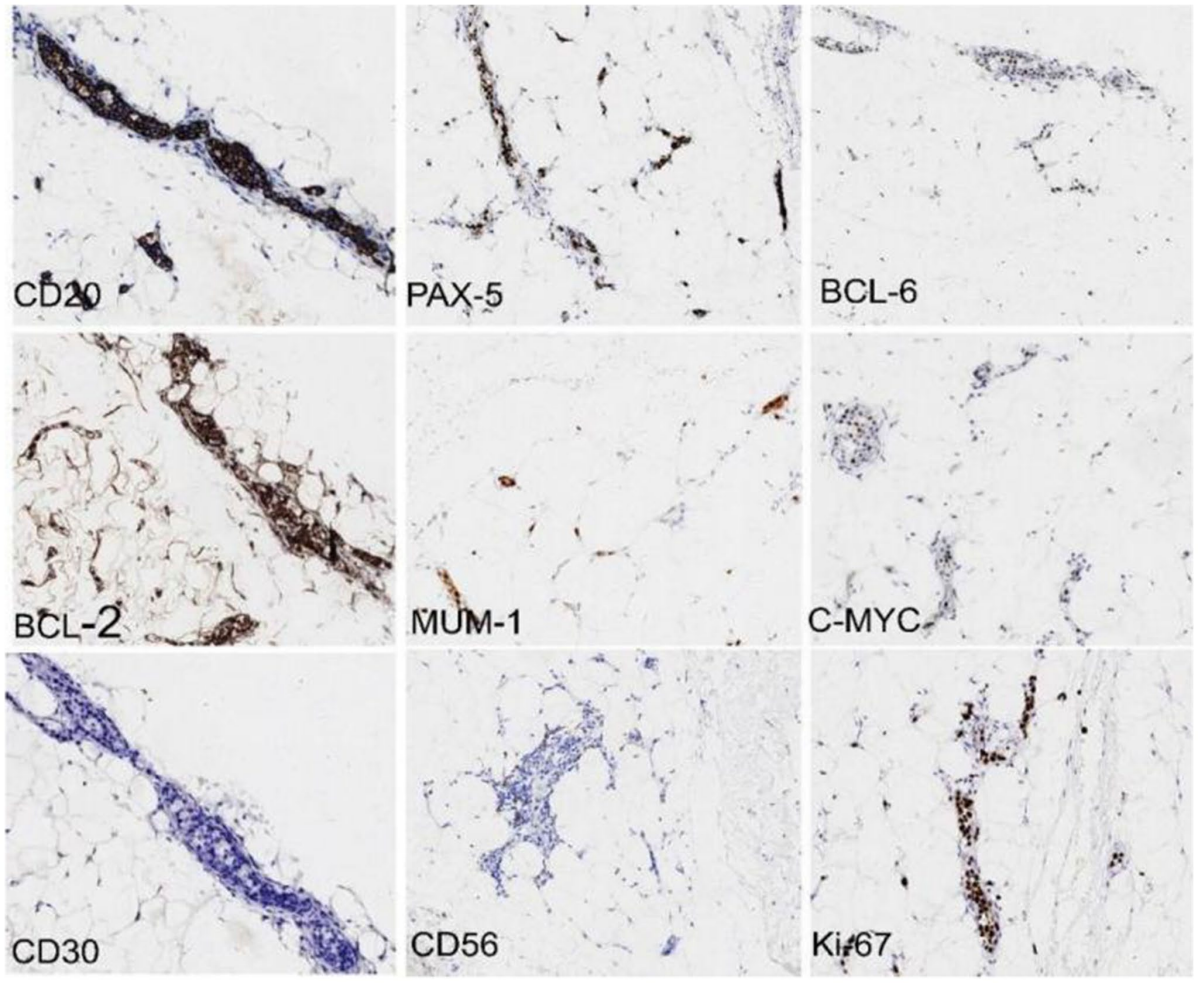
Immunohistochemistry (10X magnification) revealed that the medium to medium-large atypical lymphocytes in the blood vessels were CD20 (+), PAX-5 (+), BCL-2 (+, 80%), BCL-6 (+), MUM-1 (+), C-MYC (+, 40%), CD3 (−), CD10 (−), and Ki67 (+, 90%). The pathological diagnosis was mature B-cell lymphoma, consistent with diffuse large B-cell lymphoma (intravascular large B-cell lymphoma). Immunohistochemical Hans classification suggests that it may be of a non-germinal center B-cell origin. The immunophenotype indicates double expression of BCL-2 and C-MYC (World Health Organization classification: aggressive).

Further examinations were carried out to improve the diagnosis. A peripheral blood test for interleukin (IL)-10 and antineutrophil cytoplasmic antibody (ANCA) revealed an IL-10 level of >1000.0 pg/ml (reference: <9.1) and negative results for P-ANCA and C-ANCA. On March 5^th^, 2024, three spindle-shaped skin tissues were randomly taken from the abdomen and the medial sides of both thighs for pathological examination. The three skin tissues submitted for examination had similar morphological features, and there was no atypical epidermal hyperplasia. In the subcutaneous adipose tissue, medium to medium-large atypical lymphocytes were observed infiltrating the blood vessels. The nuclear to cytoplasmic ratio was high, and some nucleoli were visible. Immunohistochemical analysis of sample No. 2 identified the medium to medium-large atypical lymphocytes in the blood vessels as CD20 (+), PAX-5 (+), CD10 (−), BCL-6 (+), MUM-1 (+), BCL-2 (+, 80%), C-MYC (+, 40%), Ki67 (+, 90%), CD3 (−), CD5 (−), CD4 (−), CD8 (−), CD56 (−), CD7 (−), CK (−), and CD30 (+, <1%). The pathological diagnosis was mature B-cell lymphoma, consistent with diffuse large B-cell lymphoma (IVLBCL). Immunohistochemical Hans classification suggested it might be of non-germinal center B-cell origin. The immunophenotype suggested double expression of BCL-2 and C-MYC (World Health Organization classification: aggressive; Figures 2, 3). Fluorescence *in situ* hybridization examination was negative for BCL-2 and C-MYC XR1 (p. S459N); 4. PAX5 (p.*392 W); 5. CIITA (p. P146S). The revised diagnosis was stage IV IVLBCL (non-germinal center B-cell biphenotype) with CNS involvement (aaIPI score: 3 points, high risk).

The first course of chemotherapy, using the R-CHOP regimen (CTX 750 mg/m^2^ D1, epirubicin 70 mg/m^2^ D1, V 1.4 mg/m^2^ [max 2 mg] D1, dexamethasone 10 mg from day 1 to day 5). Rituximab (375 mg/m^2^ D6) was administered from March 9^th^, 2024. Orelabrutinib (150 mg/day) was added on March 14^th^. A lumbar puncture was performed once every 3 weeks, and methotrexate (MTX, 10 mg), cytarabine (50 mg), and dexamethasone (10 mg) were injected intrathecally. The patient’s Padua prediction score was in the low-risk group and no preventive anticoagulation therapy was taken. After the first course of chemotherapy was complete, the patient’s body temperature returned to normal, and pain in both lower extremities, as well as the stinging pain during urination, disappeared, and the numbness in both lower extremities did not worsen. Blood indicator re-examination on April 1^st^, 2024, revealed white blood cell, hemoglobin, platelet, and IL-10 levels of 6.6 × 10^9^/L, 84 g/L, 329 × 10^9^/L, and < 5.0 pg/ml, respectively. Biochemical tests revealed ALB and LDH levels of 34.1 g/L and 175 U/L, respectively. Starting from April 2^nd^, three courses of high-dose MTX regimen (3 g/m^2^, since the patient’s methylenetetrahydrofolate reductase gene c.677C > T polymorphism result was C/T type, the MTX dose was reduced to 2/3) chemotherapy + R-CHOP (the same dose as before) were administered, once every 3 weeks. The patient did not have severe infections during the chemotherapy and was discharged from the hospital as planned after chemotherapy completion. A schematic of the patient’s timeline information is shown in Supplementary Figure 4.

The patient visited several hospitals from illness onset until a definite diagnosis was made, and it took 8 months to get diagnosed. After that, formal treatment effectively controlled the condition. Unfortunately, because of financial reasons, the patient had to interrupt chemotherapy, but continued to take oral orelabrutinib outside the hospital. As of October 18^th^, 2024, a telephone follow-up 34 weeks after discharge revealed that the patient had a normal body temperature, and the numbness and pain in the lower extremities had improved with the help of rehabilitation acupuncture treatment.

## 3 Discussion

IVLBCL is distinguished by the congregation of a substantial number of tumor cells in the lumens of small- to medium-sized blood vessels. It has a propensity to infiltrate the nervous system, skin, bone marrow, and lungs, and lymph nodes are less commonly affected. About 30–40% of patients present with CNS symptoms, predominantly focal neurological deficits resulting from cerebral circulation disorders. Other organ involvement manifestations include renal and adrenal insufficiency, pulmonary hypertension, hypoxemia, pulmonary embolism, or respiratory failure (5).

Patients with involved IVLBCL exhibit a wide range of clinical manifestations, which depend on the affected sites. PET-CT can detect the involvement of intravascular large B-cell lymphoma in multiple important organs of the body, which helps to detect the scope and extent of the lesion at an early stage and plays an important role in assisting pathological sampling. However, it is worth noting that a negative PET-CT result cannot rule out IVLBCL (6, 7). In this case, the patient’s spinal cord was affected, and disease onset was characterized by cauda equina syndrome. The clinical manifestations were as follows: (1) radiating pain in both lower extremities, (2) nerve damage (sensory impairment mainly manifested as perineal sensory impairment, and motor impairment mainly exhibited as decreased muscle strength in the lower extremities and paralysis in severe cases), (3) sphincter dysfunction, which manifested as urination difficulty, urinary retention, and fecal and urinary incontinence. A plain MRI scan of the entire spinal cord conducted at an external hospital in October 2023 indicated a significant enhancement of the spinal meninges surrounding the conus medullaris and cauda equina. IVLBCL with spinal cord involvement as the initial manifestation is exceedingly rare, and a PUBMED search for “IVLBCL” and “CNS” retrieved only a few case reports (8, 9).

The patient underwent lumbar puncture on multiple occasions throughout the course of the disease, and the results demonstrated an increase in quantitative protein, yet CSF flow cytometry results were negative each time. This is probably because of tumor cell proliferation within the blood vessels. Consequently, cytological CSF examination typically yields negative results, which further complicates the diagnosis of the disease. Research indicates that at serum IL-10 levels of >95.65 pg/ml, IVLBCL diagnostic sensitivity and specificity are 80 and 100%, respectively, and serum IL-10 levels can also be used to monitor therapeutic effects (10). In this patient, the serum IL-10 level was >1,000.0 pg/ml before treatment, but it reduced to <5.0 pg/ml following one course of chemotherapy with the CHOP regimen. Therefore, serum IL-10 levels can be used as one of the indicators for the evaluation and monitoring of treatment effects of IVLBCL.

The diagnosis of IVLBCL is mainly based on histopathology, that is, the presence of large B-lymphoma cells in the lumen of small to medium-sized blood vessels. Bone marrow or deep skin biopsy is usually recommended as the sampling site (11, 12). Detection of cytokines such as IL-10/IL-6 plays an important role in assisting the diagnosis and treatment of lymphomas in special locations (13). IVLBCL is difficult to differentiate from Diffuse large B-cell lymphoma (DLBCL) involving blood vessels and lymphatic vessels. DLBCL sometimes forms tumor thrombi in the cavities of blood vessels and lymphatic vessels, accompanied by obvious extravascular masses, such as lymph node involvement and enlargement or extranodal organ occupying lesions. It needs to be differentiated based on clinical history, imaging examination and histopathological diagnosis.

IVLBCL prognosis depends on the sites it involves. Patients with skin involvement only usually have a relatively better prognosis. Those showing CNS involvement and complicated with hemophagocytic syndrome at the time of diagnosis have a poor prognosis. It is also clearly related to whether the patient receives treatment or not. Being >60 years old, not receiving high-dose methotrexate, and failing to achieve complete remission are adverse prognostic factors (4). In this case, the lymphoma involved the CNS, and the prognosis was poor. Research has demonstrated that for CNS-involving diffuse large B-cell lymphoma, the enhancement of patient prognosis remains restricted even with intrathecal injection and chemotherapy using the HD-MTX + CHOP regimen (14). The PRIMEUR-IVL (15) study revealed that for IVLBCL patients without overt CNS involvement, who were treated with R-CHOP + HD-MTX chemotherapy along with intrathecal injection chemotherapy and followed up for a median of 3.9 (2.5–5.5) years, the two-year PFS rate was 76%, and the risk of CNS recurrence within 2 years was 3%. Takuhei Murase et al. (16) analyzed 81 patients diagnosed with IVLBCL (79 with survival data) and found that among seven patients who received high-dose chemotherapy supplemented with autologous stem cell transplantation as consolidation treatment, five patients remained alive without recurrence after follow-up for 10.5, 18.5, 29, 39, and 95.5 months after diagnosis.

This patient had the classic subtype of IVLBCL involving the CNS. IVLBCL exhibits the characteristics of activated B-cell-type DLBCL, and MYD88 (57%), CD79B (67%), and SETD1B (57%) gene mutations are highly frequent (11). It has a considerably poorer prognosis than nodular DLBCL. Next-generation sequencing of target genes in this patient’s tumor tissue detected gene variations, such as BTG2 (p.s110fs), PRDM1 (p.s276*), NOTCH2 (p.y2340S), ITPKB (p.a485v), TBL1XR (p.s459N), PAX5 (p.*392 W), and CIITA (p.p146S), which implicate nuclear factor kappa-light-chain-enhancer of activated B-cells (NF-κB), NOTCH, ITPKB, Wnt/β-catenin, and EGFR signaling pathways. The BTG2 mutation facilitates p65’s entry into the nucleus and activates NF-κB signaling, which impacts the molecular mechanism underlying DLBCL’s malignant phenotype. PRDM1 and PAX5 mutations are also engaged in NF-κB signaling modulation. The B-cell receptor/NF-κB signaling axis sustains malignant B-cell survival and proliferation (17). BTK is primarily involved in the B-cell receptor/NF-κB signaling pathway, and BTK inhibitors have emerged as promising drugs for B-cell lymphoma (18). Orelabrutinib, a highly selective new-generation BTK inhibitor, can permeate the blood–brain barrier and has a high CSF/plasma ratio. The most prominent distinction between orelabrutinib and other BTK inhibitors is its central skeleton’s monocycle structure, which enhances target selectivity and diminishes off-target toxicity.

Studies have demonstrated that BTK inhibitors can enhance the survival of patients with lymphoma and CNS involvement (19, 20), and orelabrutinib can effectively and safely treat primary CNS lymphoma (21). Therefore, we incorporated orelabrutinib into R-CHOP + HD-MTX and intrathecal medication to augment the therapeutic effect, which can be considered a novel treatment approach. Currently, there is no clinical evidence of orelabrutinib use to treat CNS-IVLBCL. In this patient, the treatment efficacy of combining orelabrutinib and the relatively favorable treatment outcome might provide a proof-of-principle for IVLBCL patients. Future studies should include a larger number of patients with this condition to explore new treatment strategies.

## Data Availability

The datasets presented in this study can be found in online repositories. The names of the repository/repositories and accession number(s) can be found in the article/Supplementary material.

## References

[1] Turner JJ Morton LM Linet MS Clarke CA Kadin ME Vajdic CM. Interlymph hierarchical classification of lymphoid neoplasms for epidemiologic research based on the who classification (2008): update and future directions. Blood. (2010) 116:e90–8. doi: 10.1182/blood-2010-06-289561, PMID: 20699439 PMC2993636

[2] Alaggio R Amador C Anagnostopoulos I Attygalle AD de Oliveira Araujo IB Berti E. International Agency for Research on Cancer/World Health Organization correction: "the 5th edition of the World Health Organization classification of Haematolymphoid Tumours: lymphoid neoplasms" leukemia. Leukemia. (2022) 37:1944–51. doi: 10.1038/s41375-023-01962-5, PMID: 37468552 PMC10457187

[3] Ponzoni M Arrigoni G Gould VE Del Curto B Maggioni M Scapinello A. Lack of cd 29 (beta1 integrin) and cd 54 (Icam-1) adhesion molecules in intravascular lymphomatosis. Hum Pathol. (2000) 31:220–6. doi: 10.1016/S0046-8177(00)80223-3, PMID: 10685637

[4] Kasuya A Fujiyama T Shirahama S Hashizume H Tokura Y. Decreased expression of homeostatic chemokine receptors in intravascular large B-cell lymphoma. Eur J Dermatol. (2012) 22:272–3. doi: 10.1684/ejd.2012.1639, PMID: 22381519

[5] Geer M Roberts E Shango M Till BG Smith SD Abbas H. Multicentre retrospective study of intravascular large B-cell lymphoma treated at academic institutions within the United States. Br J Haematol. (2019) 186:255–62. doi: 10.1111/bjh.15923, PMID: 31044423 PMC8989046

[6] Schönau V Vogel K Englbrecht M Wacker J Schmidt D Manger B. The value of (18)F-Fdg-pet/Ct in identifying the cause of fever of unknown origin (Fuo) and inflammation of unknown origin (Iuo): data from a prospective study. Ann Rheum Dis. (2018) 77:70–7. doi: 10.1136/annrheumdis-2017-211687, PMID: 28928271

[7] Matsukura K Hokkoku K Shiraoka A Yang L Takahashi Y Hatanaka Y. Increased uptake on (18) F-Fluorodeoxyglucose positron emission tomography/computed tomography is indicative of occult skin lesions in a patient with intravascular large B-cell lymphoma. J Dermatol. (2018) 45:e254–5. doi: 10.1111/1346-8138.14302, PMID: 29569289

[8] Vandermeersch D Mahsouli A Willemart M Scoppettuolo P Van de Wyngaert C Van den Neste E. Intravascular large cell B lymphoma presenting as central nervous system Pseudo-Vasculitis: A rare diagnostic challenge. Neuroradiol J. (2024) 37:651–5. doi: 10.1177/19714009231212351, PMID: 37933603 PMC11444320

[9] Terao T Tsushima T Ikeda D Fukumoto A Kamura Y Kuzume A. Limited efficacy of high-dose methotrexate to prevent the central nervous system relapse in patients with Ivlbcl. Leuk Lymphoma. (2022) 63:3394–401. doi: 10.1080/10428194.2022.2123239, PMID: 36111741

[10] Zhang Y Wang L Sun J Wang W Wei C Zhou D. Serum Interleukin-10 as a valuable biomarker for early diagnosis and therapeutic monitoring in intravascular large B-cell lymphoma. Clin Transl Med. (2020) 10:e131. doi: 10.1002/ctm2.131, PMID: 32634257 PMC7418806

[11] Shimada K Yoshida K Suzuki Y Iriyama C Inoue Y Sanada M. Frequent genetic alterations in immune checkpoint-related genes in intravascular large B-cell lymphoma. Blood. (2021) 137:1491–502. doi: 10.1182/blood.2020007245, PMID: 33512416 PMC7976508

[12] Narimatsu H Morishita Y Saito S Shimada K Ozeki K Kohno A. Usefulness of bone marrow aspiration for definite diagnosis of Asian variant of intravascular lymphoma: four autopsied cases. Leuk Lymphoma. (2004) 45:1611–6. doi: 10.1080/10428190410001683769, PMID: 15370213

[13] Song Y Zhang W Zhang L Wu W Zhang Y Han X. Cerebrospinal fluid Il-10 and Il-10/Il-6 as accurate diagnostic biomarkers for primary central nervous system large B-cell lymphoma. Sci Rep. (2016) 6:38671. doi: 10.1038/srep38671, PMID: 27924864 PMC5141427

[14] Liu Z Zhang Y Zhu Y Zhang W. Prognosis of intravascular large B cell lymphoma (Ivlbcl): analysis of 182 patients from global case series. Cancer Manag Res. (2020) 12:10531–40. doi: 10.2147/cmar.S267825, PMID: 33122951 PMC7591067

[15] Shimada K Yamaguchi M Atsuta Y Matsue K Sato K Kusumoto S. Rituximab, cyclophosphamide, doxorubicin, vincristine, and prednisolone combined with high-dose methotrexate plus intrathecal chemotherapy for newly diagnosed intravascular large B-cell lymphoma (Primeur-Ivl): A multicentre, single-arm, phase 2 trial. Lancet Oncol. (2020) 21:593–602. doi: 10.1016/S1470-2045(20)30059-0, PMID: 32171071

[16] Murase T Yamaguchi M Suzuki R Okamoto M Sato Y Tamaru J. Intravascular large B-cell lymphoma (Ivlbcl): A Clinicopathologic study of 96 cases with special reference to the Immunophenotypic heterogeneity of Cd5. Blood. (2007) 109:478–85. doi: 10.1182/blood-2006-01-021253, PMID: 16985183

[17] Robak T Witkowska M Smolewski P. The role of Bruton's kinase inhibitors in chronic lymphocytic leukemia: current status and future directions. Cancers (Basel). (2022) 14:771. doi: 10.3390/cancers14030771, PMID: 35159041 PMC8833747

[18] McDonald C Xanthopoulos C Kostareli E. The role of Bruton's tyrosine kinase in the immune system and disease. Immunology. (2021) 164:722–36. doi: 10.1111/imm.13416, PMID: 34534359 PMC8561098

[19] Rusconi C Cheah CY Eyre TA Tucker D Klener P Giné E. Ibrutinib improves survival compared with chemotherapy in mantle cell lymphoma with central nervous system relapse. Blood. (2022) 140:1907–16. doi: 10.1182/blood.2022015560, PMID: 35789260

[20] Cheng Q Wang J Lv C Xu J. Successful Management of a Patient with refractory primary central nervous system lymphoma by Zanubrutinib. Onco Targets Ther. (2021) 14:3367–72. doi: 10.2147/OTT.S309408, PMID: 34079282 PMC8163630

[21] Shen J Liu J. Bruton's tyrosine kinase inhibitors in the treatment of primary central nervous system lymphoma: A Mini-review. Front Oncol. (2022) 12:1034668. doi: 10.3389/fonc.2022.1034668, PMID: 36465385 PMC9713408

